# Alternative Balance between Transcriptional and Epigenetic Regulation during Developmental Proliferation of Human Cranial Neural Crest Cells

**DOI:** 10.3390/cells13191634

**Published:** 2024-09-30

**Authors:** Christophe Desterke, Raquel Francés, Claudia Monge, Agnès Marchio, Pascal Pineau, Jorge Mata-Garrido

**Affiliations:** 1 Faculté de Médecine du Kremlin Bicêtre, Université Paris-Saclay and INSERM UMRS1310, 94270 Le Kremlin-Bicêtre, France; christophe.desterke@inserm.fr; 2 Cell and Tissue Biology Group, Anatomy and Cell Biology Department, University of Cantabria-IDIVAL, 39011 Santander, Spain; 3 International Joint Laboratory of Molecular Anthropological Oncology (LOAM), IRD, INEN, Lima 15038, Peru

**Keywords:** craniofacial disease, epigenetics, neural crest, development, embryogenesis

## Abstract

Cranial neural crest cells are implicated in multiple transcriptional events at the different stages of differentiation during development. The alteration of some transcription factors expressed during neural crest development, like PAX7, could be implicated in the etiology of face malformation in murine models. Epigenetic regulation has been shown to be an important mechanistic actor in the control of timing and the level of gene expression at different stages of neural crest development. During this work, we investigated the interconnection between epigenetics and transcription factors across a diversity of human development cranial neural crest cells. Across a diversity of neural cells from human developing cranial tissues, in accordance with their proliferation stage, an alternative balance of regulation between transcription factors and epigenetic factors was identified.

## 1. Introduction

Nervous system development is a process of extraordinary complexity that begins very early in embryogenesis and continues into adult life. This process involves a series of coordinated cellular and molecular events that result in the formation of the brain, spinal cord, and peripheral nervous system [1,2,3]. Within this framework, neural crest cells (NCCs) play a crucial role due to their unique ability to generate a diversity of cell types through migration and differentiation processes [4,5]. The neural tube, derived from the neurectoderm (a specialized region of the ectoderm), extends along the anteroposterior axis of the embryo. Neural crest cells (NCCs) originate from the neural tube and are formed along its entire length. These cells contribute to the development of various tissues in both the anterior (brain) and posterior (spinal cord) regions of the embryo [4]. One of the most remarkable features of NCCs is their ability to differentiate into multiple lineages. Depending on their axial origin (cranial, vagal, truncal, or sacral), NCCs can generate a wide range of cell types. Cranial neural crest cells (CNCCs) differentiate into neurons and glia of the cranial ganglia, Schwann cells, melanocytes, odontoblasts, chondrocytes, and osteoblasts, which contribute to the formation of bones and cartilage in the skull and face. Vagal and sacral NCCs generate neurons of the enteric nervous system, while truncal NCCs produce neurons and glia of the peripheral nervous system, Schwann cells, and melanocytes [4]. In this article, we will focus specifically on CNCCs.

The development and differentiation of CNCCs are governed by a complex interplay between transcriptional and epigenetic regulatory mechanisms. These two layers of regulation work together to ensure that cells follow the right developmental pathways at the right time and in the right place. Understanding these mechanisms is critical to unraveling how CNCCs achieve their remarkable plasticity and ability to differentiate into multiple cell types.

### 1.1. Transcriptional Regulation

Transcriptional regulation involves the control of gene expression at the transcriptional level, mediated by transcription factors that bind to specific DNA sequences [6,7]. These factors can act as activators or repressors of transcription and often work in combination to control networks of genes involved in specific developmental processes. CNCC development is known to be controlled by an interplay of several transcription factors [8,9,10].

### 1.2. Epigenetic Regulation

Epigenetic regulation includes, among other aspects, DNA and histone modifications that affect the accessibility of DNA to the transcriptional machinery without altering the DNA sequence [11,12]. These epigenetic mechanisms play a crucial role in the long-term regulation of gene expression, influencing a wide range of biological processes, including the regulation of the cell cycle, cell differentiation, and cell fate determination, among others [11]. In this article, we highlight the significance of DNA methylation, which involves the methylation of cytosine residues in DNA and is typically linked to transcriptional repression. We also emphasize DNA hydroxymethylation, where methylcytosine is converted to hydroxymethylcytosine by TET proteins, a process that may be crucial for epigenetic reprogramming during development [13,14]. In addition, histones, which together with DNA form part of the chromatin, are susceptible to epigenetic changes, such as histone acetylation, which is generally associated with transcriptional activation as it relaxes the chromatin structure and allows for easier access of the transcriptional machinery to the DNA (in CNCCs, histone acetylation can regulate the expression of genes required for migration and differentiation [15,16]), or histone methylation, a modification whose activity depends on the location and context and can be associated with both the activation and the repression of transcription [17].

However, transcriptional and epigenetic regulation are not independent processes; they interact in a dynamic and coordinated manner. Transcription factors can recruit epigenetic enzymes to the promoters and enhancers of specific genes to modify chromatin and regulate gene expression. For example, Sox10 can recruit histone acetyltransferases (HATs) to activate the transcription of target genes [18], whereas Snail and Slug can recruit histone deacetylases (HDACs) to repress the transcription of genes that inhibit migration [19].

Importantly, during embryonic development, CNCCs undergo a series of carefully regulated processes of proliferation, migration, and differentiation to form various craniofacial structures. These processes are driven by extrinsic and intrinsic signals that interact to ensure that the CNCCs perform their function properly. Understanding these processes is essential to unravel developmental mechanisms and address neural crest-related pathologies [20,21,22]. The proliferation of CNCCs is a critical process that ensures a sufficient number of progenitor cells for the formation of complex craniofacial structures. This regulation of proliferation involves several growth factors, such as the epidermal growth factor (EGF), fibroblast growth factor (FGF), and insulin-like growth factor (IGF) [23], and signals that modulate the cell cycle, such as the PI3K/Akt pathway, MAPK/ERK pathway, and Notch pathway [24,25].

The proliferation of CNCCs is tightly controlled by the cell cycle machinery, which includes positive and negative regulators. Cyclins and CDKs form complexes that drive the cell through the different phases of the cell cycle. On the other hand, CDK inhibitors (CKIs) such as p21 and p27 can slow down the cell cycle and allow cells to enter a state of quiescence or differentiation [26,27]. Once this initial proliferation process is completed, the CNCCs must migrate to their specific destinations in the embryo. This event is crucial for the formation of craniofacial structures and depends on precise signals that guide the cells through complex tissues. There are two fundamental processes involved in these migration processes: the epithelial–mesenchymal transition (EMT), where epithelial cells acquire mesenchymal characteristics that allow them to migrate [28,29], and interactions with extracellular matrix (ECM) components such as fibronectin, laminin, and collagen, a process with the ability to direct migration [30,31].

Alterations in the proliferation, migration, and differentiation of CNCCs can result in craniofacial malformations, neurocristopathic diseases, and cancers such as neuroblastoma and melanoma [32,33,34,35,36,37].

During this work at the single-cell level, we investigated the interplay between the expression of epigenetic regulators and transcription factors across the differentiation and proliferation stages of human CNCCs.

## 2. Materials and Methods

### 2.1. Public Datasets

The scRNA-seq Seurat object from GSE197513, containing a quantification of 28,470 features for 4233 human developing cranial neural crest cells (CNCCs), was downloaded from the Gene Expression Omnibus (GEO) website [38]. This Seurat object, pre-processed, normalized, scaled, and annotated by the original authors [39], was loaded into R using the RDS file format.

### 2.2. General Bioinformatics Analyses

All bioinformatics analyses were conducted using the R software environment, version 4.3.3, along with Bioconductor version 3.18 [40]. The complete R code for these analyses is available at the following address: bioinformatics code repository (https://github.com/bioinform; accessed on 18 July 2024).

### 2.3. Single-Cell RNA Sequencing Data Preparation

After loading the original single-cell experiment object into R, a subset limited to neural cells was created using the Seurat R package, version 5.1.0 [41]. The standard Seurat pipeline, including normalization, the identification of variable features, and scaling, was then applied to this neural cell subset, which contained 173 samples.

### 2.4. Single-Cell RNA Sequencing Cell Trajectory Analysis

After converting the neural cell Seurat object into a SingleCellExperiment object, a cell trajectory was constructed using two different approaches: the Slingshot R-Bioconductor package, version 2.10.0 [42], and the TSCAN R-Bioconductor package, version 1.40.1 [43,44]. To determine the root of the trajectory, cell entropy was computed using the TSCAN function “perCellEntropy” [45,46].

### 2.5. Single-Cell Geneset Activity

The geneset activity for each cell was determined using a subsection of the human cranial neural crest cell (CNCC) differentiation pathway (WP2064), available at Wikipathways (https://www.wikipathways.org/; accessed on 18 September 2024). This pathway includes several subsections: epidermis, neural plate border, premigratory CNCC, migratory CNCC, mesoderm, neuron/glia-autonomous nervous system, chondrocytes, and melanocytes [47]. After reconstituting the geneset, single-cell activity was quantified using the escape R-Bioconductor package, version 1.12.0 [48], employing the gene set variation analysis (GSVA) metric, version 1.50.5 [49]. Data visualizations for single-cell analyses were primarily generated using the scater R-Bioconductor package, version 1.30.1 [50].

### 2.6. Co-Expression Network and Connectivity Analyses of scRNA-Seq Neural Cells

Transcription factors [51] and epigenetic factors (EpiFactors) [52] were extracted from the significant genes identified along the pseudotime trajectory of neural cells (Table S1, DOI: 10.6084/m9.figshare.26334847). By associating the expression profiles of these transcription factors and epigenetic factors, a matrix was constructed for network co-expression analysis using the Weighted Gene Co-expression Network Analysis (WGCNA) method [53,54], executed with the WGCNA R package, version 1.72-5. This included the construction of an adjacency matrix and module identification for the transcription/epigenetic factors matrix. Cell phenotype–trait correlations were performed for the four identified modules: blue, yellow, turquoise, and brown. For each module, connectivity was quantified for enriched epigenetic factors and transcription factors. Hub markers were identified based on connectivity projections within each module, with network visualizations generated using the igraph R package, version 2.0.3 [55].

## 3. Results

### 3.1. High Cell Entropy in Human Developing Proliferative Cranial Neural Crest Cells

**Figure 1:**
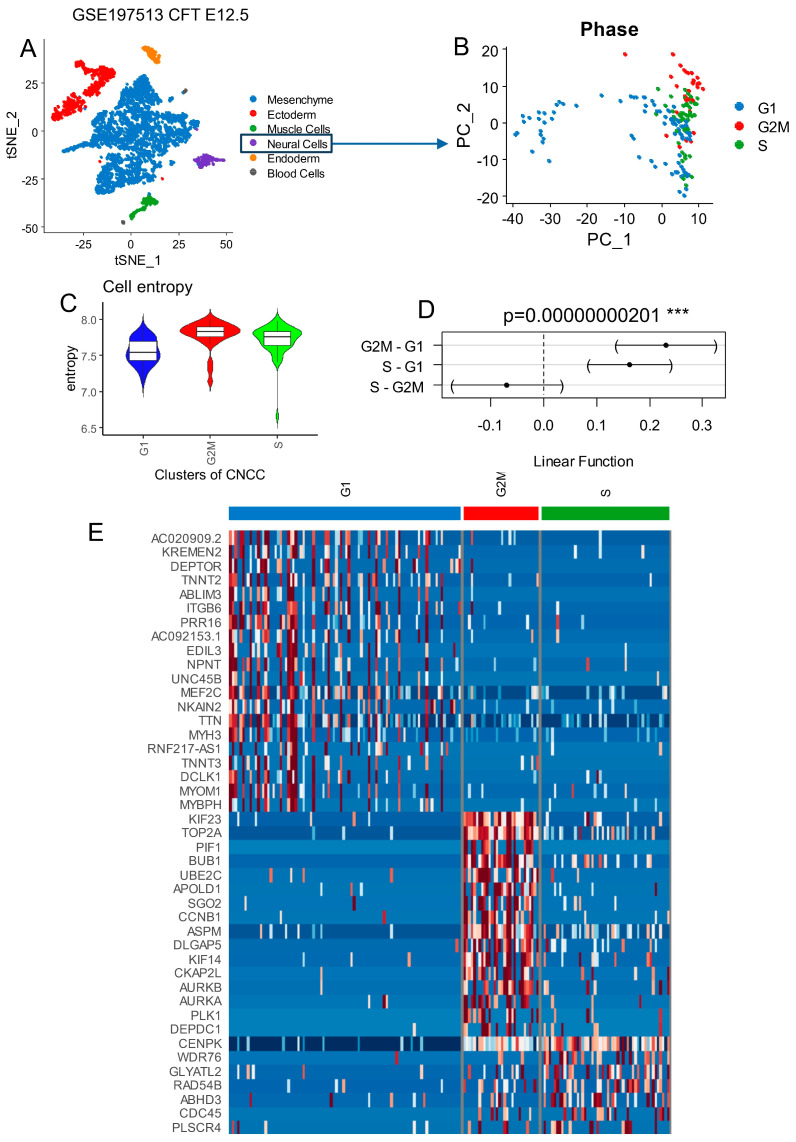
Cell cycle phase decomposition of neural cells from developing human face tissues: human face (Carnegie Stage CS17) tissue from scRNAseq dataset GSE197513. (**A**) scRNAseq tSNE from all cell subtypes from human developing face tissues. (**B**) Principal component analysis for dimension reduction of scRNAseq from neural cells according to cell cycle phase projection. (**C**) Cell entropy performed on neural cells according to cell cycle phase stratification. (**D**) One-way ANOVA with Tukey post hoc tests performed for cell entropy according to cell cycle phases as the outcome. (**E**) Heatmap of best markers identified in scRNAseq of developing face tissues according to the different phases of cell cycles characterized in neural cell subtypes.

Single-transcriptome RNA sequencing from human developing cranial face tissue (Carnegie Stage (CS) 17) was investigated with the Seurat pipeline and original cell annotation of the authors (GEO dataset GSE197513) [39]. Carnegie Stage 17 of human development (41–43 days post-fertilization) features an embryo measuring about 11–14 mm, with more pronounced limb buds, emerging digital rays, and distinct facial structures like the developing ears and deepening nasal pits. The heart is structurally advanced, the brain and spinal cord are growing, and somites (about 41–44 pairs) are present. Organ systems like the digestive, nervous, and genitourinary systems continue differentiating, with the primitive gut and genital ridges becoming more distinct [56]. After principal component analysis, a t-SNE dimension reduction (Figure 1A) allowed a proper stratification of the distinct cell subtypes among these 4233 samples. With the Seurat package, a new single -object was built and preprocessed by sub-setting the neural cell subtype comprising 173 samples (Figure 1B). After cell cycle phase prediction [57], principal component analysis dimension reduction succeeded in stratifying the distinct cell cycle phases: G1, S, and G2M on the first principal map representative of whole-transcriptome heterogeneity of the neural cells (Figure 1B). Seurat clustering identified four distinct clusters among neural cells (Figure S1A), with distinct cell cycle phase proportions (chi-square test *p*-value < 2.2 × 10^−16^) (Figure S1B): Cell Clusters 0 and 3 were found with similar cell phase proportions (enriched in G1 and S phases), Cluster 1 was enriched in G2/M cell cycle phases, and Cluster 2 was enriched in the G1 phase. Effectively, the top specific markers of Cluster 1 in G2/M phases were enriched in proliferative markers such as TOP2A (DNA topoisomerase II alpha), CDK1 (cyclin-dependent kinase 1), CDC25C (cell division cycle 25C), AURKB (aurora kinase B), and CCNB1 (cyclin B1) (Figure S1C). These results suggest a close link between proliferation and transcriptional regulation in human developing CNCCs. The cell entropy in the single-cell RNAseq was relative to the diversity of quantification of the different transcripts inside the cells. According to Waddington’s epigenetic theory [58], cells lose transcriptional diversity as they differentiate, resulting in a less diverse transcriptional program compared to the original stem cells. Consequently, their transcriptional program exhibits lower entropy than that of stem cells. Stem cells need to maintain high entropy in their transcriptome (high diversity of transcript quantification) in order to be capable of multipotency [45]. Per cell transcriptional diversity is an important marker for stem cell and developmental stage characterizations [45,46]. Neural cells in G2/M phases presented higher quantification levels of per cell entropy (Figure 1C), and significantly higher entropy was found in G2/M and S phases compared to the G1 phase by the Fisher test (*p*-value = 2.01 × 10^−9^, Figure 1D) followed by the Tukey post-hoc test (*p*-value < 0.001, Figure 1D). Top marker genes identified in G2/M neural cells were confirmed to be proliferative markers (Figure 1E) (TOP2A, BUB1 (BUB1 mitotic checkpoint serine/threonine kinase), CCNB1, AURKA (aurora kinase A), AURKB), like in Seurat cell cluster number 1 (Figure S1C).

### 3.2. Transcriptional Factors Regulated across Proliferative Cell Trajectory of CNCCs

**Figure 2:**
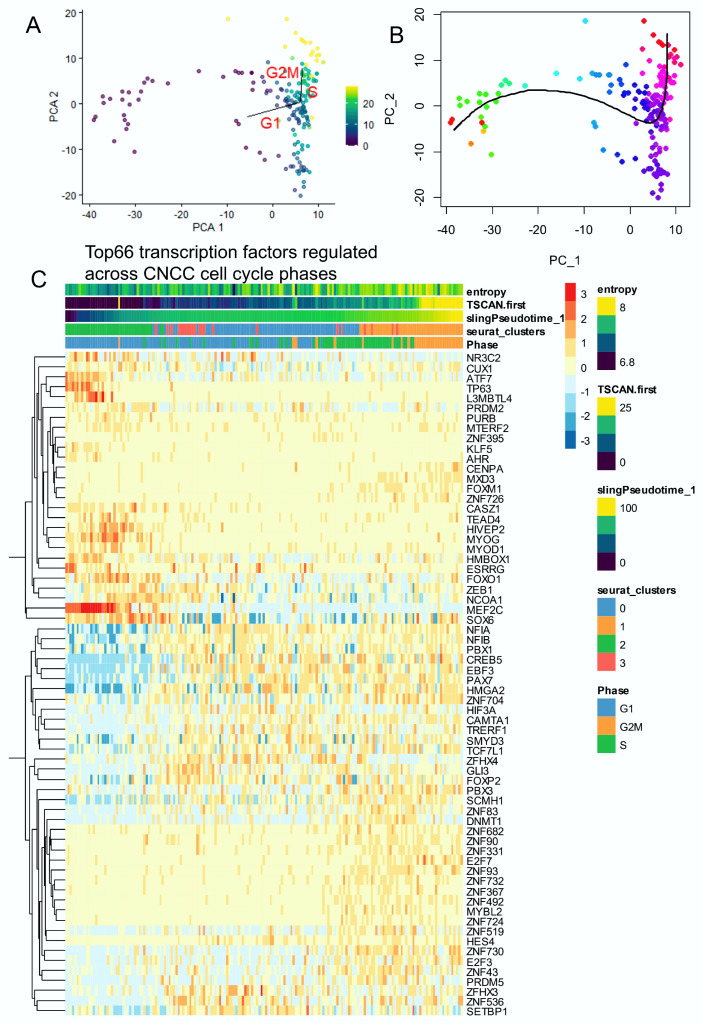
The single-cell trajectory across cell cycle decomposition of cranial neural crest cells highlighted the regulation of several transcription factors: neural cells from human (CS17) face tissue from scRNAseq dataset GSE197513. (**A**) TSCAN cell trajectory in human developing neural cells from cranial neural crest cells (CNCCs): pseudotime was quantified using principal component analysis (PCA) with cell cycle phase prediction.(color scale for TSCAN pseudotime quantification) (**B**) Slingshot pseudotime trajectory projected on PCA (color scale for Slinghot pseudotime quantification). (**C**) Heatmap of significant transcription factors (66 best markers, FDR < 0.01) identified across trajectory with cell cycle phase decomposition in human developing CNCCs.

Pseudotime quantification was performed on neural cell subtypes of murine developing cranial face tissue by two distinct algorithms (TSCAN and Slingshot, respectively Figure 2A,B). High values of single-cell pseudotime were found in G2M cells (Figure 2A), related to higher entropy (Figure 1C), which were considered, therefore, as the root of the trajectory [45]. TSCAN pseudotime analyses predicted cell cycle progression (Figure 2A) in accordance with the slingshot mono-trajectory (Figure 2B). On the TSCAN trajectory, 1183 genes were found to be significantly (False Discovery Rate <= 0.01) regulated on the proliferative trajectory of neural cells (Table S1). Among the markers for cells with the lowest pseudotime quantification were AC020909.2, TTN (titin), ITGB6 (integrin subunit beta 6), SNTB1 (syntrophin beta 1), FNDC5 (fibronectin type III domain containing 5), MEF2C (myocyte enhancer factor 2C), ACTC1 (actin alpha cardiac muscle 1), CCDC141 (coiled-coil domain containing 141), MTSS1 (MTSS I-BAR domain containing 1), and TNNT2 (troponin T2, cardiac type) (Figure S2). Among the markers for cells with the highest pseudotime quantification were TOP2A (DNA topoisomerase II alpha), PIF1 (PIF1 5′-to-3′ DNA helicase), ASPM (assembly factor for spindle microtubules), DIAPH3 (diaphanous related formin 3), BUB1 (BUB1 mitotic checkpoint serine/threonine kinase), CENPF (centromere protein F), KIF11 (kinesin family member 11), KIF14 (kinesin family member 14), TPX2 (TPX2 microtubule nucleation factor), and KIF23 (kinesin family member 23) (Figure S3). Among these 1183 pseudotime-regulated genes, 66 transcription factors were identified from the Toronto human transcription factor database [51] (Figure 2C). Single-cell expression of these 66 significantly regulated transcription factors predicted well cell entropy, TSCAN pseudotime, slingshot pseudotime, Seurat clusters, and cell cycle phases (Figure 2C). On the heatmap, it could be observed that distinct patterns of transcription factors were upregulated during the distinct phases of the CNCC cell cycle. For example, ZNF transcription factors were conspicuously expressed during S and G2M phases (Figure 2C).

### 3.3. Epifactors Regulated across Proliferative Cell Trajectory of CNCCs

**Figure 3:**
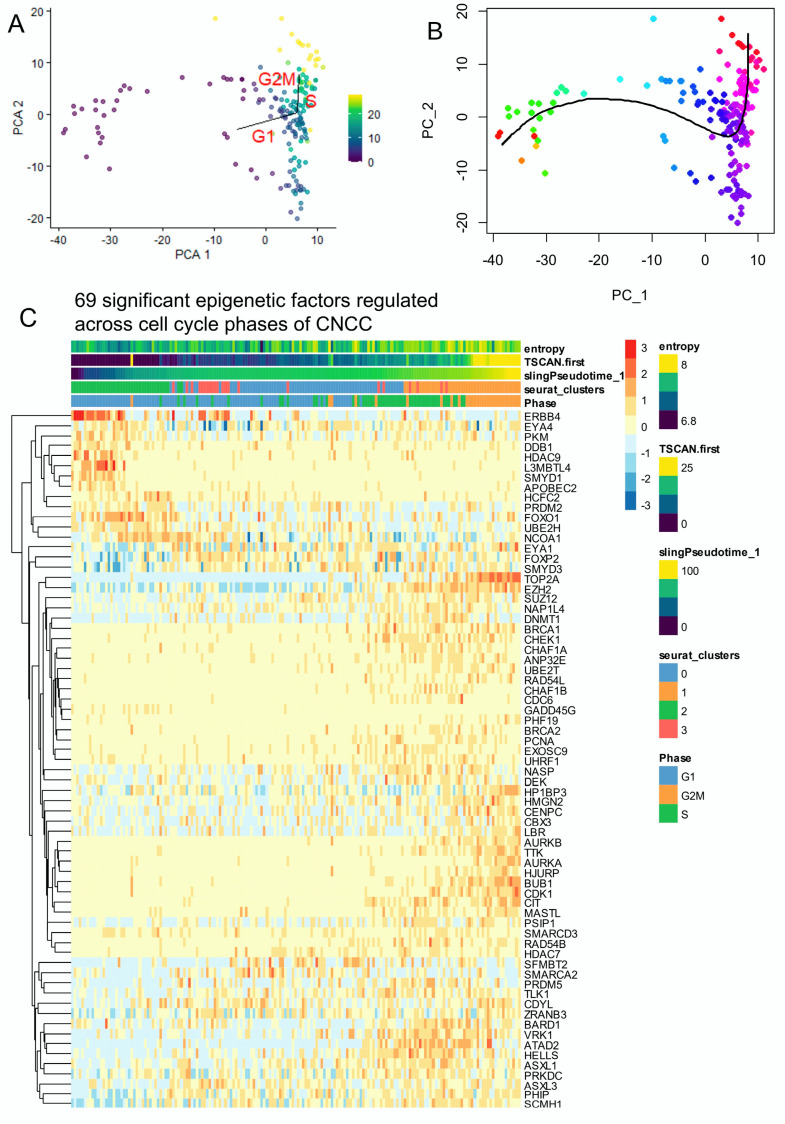
The single-cell trajectory across cell cycle decomposition of cranial neural crest cells highlighted the regulation of several epigenetics factors: neural cells from human CS17 face tissue from scRNAseq dataset GSE197513. (**A**) TSCAN cell trajectory in murine developing neural cells from cranial neural crest cells (CNCCs) (color scale for TSCAN pseudotime quantification): pseudotime was quantified using principal component analysis (PCA) with cell cycle phase prediction. (**B**) Slingshot pseudotime trajectory projected on PCA. (color scale for Slingshot pseudotime quantification) (**C**) Heatmap of significant epigenetic factors (69 best markers, FDR < 0.01) identified across trajectory with cell cycle phase decomposition in murine developing CNCCs.

Pseudotime quantification performed on neural cell subtypes of human developing cranial face tissue by two distinct algorithms (TSCAN and Slingshot) (respectively Figure 3A,B) was used again to identify EpiFactors regulated along the trajectory. Crossing significant pseudotime-regulated genes (Table S1) with the EpiFactors database [52] allowed us to identify 69 significant EpiFactors with FDR-adjusted *p*-values less than 0.01 (Figure 3C).

### 3.4. Neural Cell Trajectory Is Associated with CNCC Differentiation Processes

**Figure 4:**
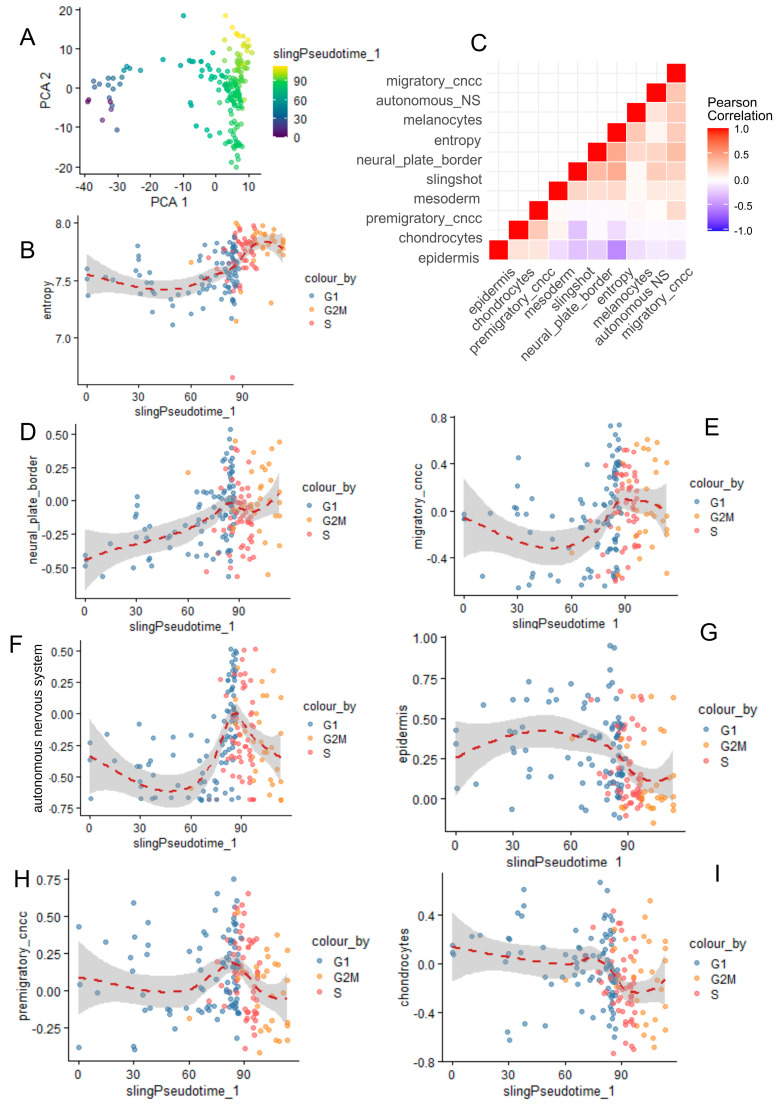
Correlation between cranial neural crest cell differentiation and single-cell trajectory/single-cell entropy across cell cycle phase decomposition. (**A**) PCA with slingshot pseudotime quantification in neural cells from CNCCs. (**B**) Scatterplot of cell entropy versus slingshot pseudotime according to cell cycle phase decomposition. (**C**) Corregram (Pearson correlation) between CNCC differentiation genesets (human wikipathway neural crest differentiation WP2064) and other variables such as entropy and slingshot pseudotime. (**D**–**I**) Representative pseudotime expression for correlated genesets and variables with cell cycle phase decomposition.

Slingshot pseudotime quantification was well stratified across the heterogeneity of the neural cell single-cell transcriptome (Figure 4A), showing higher values in the G2M phase, intermediate in the S phase, and lower in the G1 phase (Figure 4B). Single-cell geneset activity was observed for CNCC differentiation processes. A Pearson correlation analysis revealed a positive association between entropy, slingshot pseudotime, migratory CNCCs, the autonomous nervous system, melanocytes, neural plate borders, and mesoderm (Figure 4C). In contrast, chondrocyte and epidermis differentiation showed a negative correlation with these markers (Figure 4C). Pseudotime analysis confirmed a progressive relationship between cell cycle progression and neural plate border geneset activity (Figure 4D). Pseudotime association during the S phase was more associated with migratory CNCCs, the autonomous nervous system, or premigratory CNCC geneset activities (respectively, Figure 4E,F,H). Geneset activities for epidermis and chondrocyte differentiation were found to be more important during the G1 phase on the pseudotime trajectory of neural cells (Figure 4G,I). These results suggest a relation between CNCC differentiation and the proliferative cell trajectory of neural cells during murine development of cranial face tissues. Effectively, PAX7 (paired box 7) (Figure S4A) transcription factor expression during neural cell trajectory was inversely correlated to CDH15 (cadherin 15) (Figure S4A) and PIAS1 (protein inhibitor of activated STAT 1) and progressively decreased across G2M, S, and G1 cell cycle phases (Figure S4A). EpiFactors such as DNMT1 (DNA methyltransferase 1) and EZH2 (enhancer of zeste 2 polycomb repressive complex 2 subunit) were found to be particularly induced during the S phase and drastically decreased during the G1 phase (Figure S4B), like proliferative markers such as TOP2A and BUB1 (Figure S4C).

### 3.5. Alternative Regulation between Epigenetic and Transcription Factors during Proliferative Trajectory of Neural Cells from Human Developing Cranial Face Tissue

**Figure 5:**
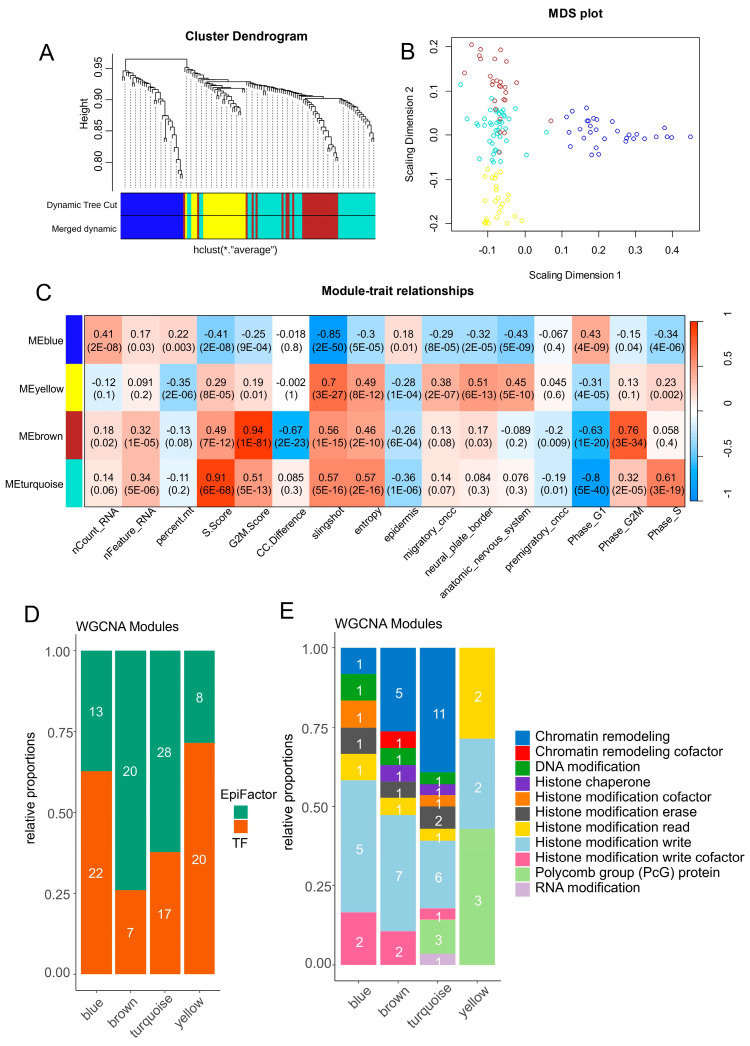
Epigenetic and transcriptional co-expression network identification in neural cell diversity of developing human CNCCs. (**A**) WGCNA gene tree clustering and module identification for selected transcription factors and EpiFactors in scRNAseq from CNCCs. (**B**) Multidimensional scaling dimension reduction for genes separated by module. (**C**) Phenotype trait correlation with gene modules identified by WGCNA analysis (color scale “blue—white—red” for Pearson correlation coefficients), numbers in cells correspond to Pearson correlation *p*-values between phenotype traits and gene modules. (**D**) Barplot of proportions and numbers for EpiFactors and transcription factors identified by gene module. (**E**) Barplot of epigenetic function classes for EpiFactors enriched by gene modules.

After the selection of single-cell expression of significant transcription and epigenetic factors (Figure 2 and Figure 3), a co-expression network analysis [53] was performed on neural cell subtypes of CNCCs. Clustering on an adjacent matrix (Figure S5A) allowed for the identification of four main gene modules (Figure 5A): a blue module containing 31 molecules, a turquoise module containing 43 molecules, a brown module containing 27 molecules, and a yellow module containing 25 molecules (Figure 5B). Phenotypic trait correlation mainly showed a significant positive relation between three modules (yellow, brown, and turquoise) and cell entropy + slingshot pseudotime quantification (Figure 5C). Inversely, the blue module was found to have a negative correlation with pseudotime and cell entropy (Figure 5C). The yellow module was found to have a significant positive correlation with CNCC differentiation genesets such as neural plate borders, the autonomous nervous system, and migratory CNCCs (Figure 5C). The blue module was positively correlated to the G1 phase and epidermis differentiation (Figure 5C). The brown and turquoise modules were found to have a significant positive correlation with the proliferative phases of the cell cycle, respectively, the G2M and S phases (Figure 5C). The brown and turquoise modules associated with the proliferative phases of cell cycles were found to be particularly enriched in genes implicated as epigenetic factors with low transcription factor enrichment (Figure 5D). Conversely, the yellow and blue modules associated with the differentiation of CNCCs harbored a higher proportion of transcription factors (Figure 5D). These results suggest an alternative balance of regulation between epigenetic and transcription factors during the proliferative trajectory of neural cells from developing murine facial tissues. When focusing on epigenetic regulation, a high proportion of molecules implicated in chromatin remodeling and histone modification was observed in cell cycle-associated modules (brown and turquoise) (Figure 5E). It is interesting to notice an epigenetic switch to polycomb member enrichment in the yellow module in positive correlation with neural plate border differentiation (Figure 5E), represented by SCMH1 (Scm polycomb group protein homolog 1), ASXL3 (ASXL transcriptional regulator 3), and SFMBT2 (Scm-like with four MBT domains 2).

### 3.6. Epigenetic Hub Regulation during Proliferative Phases of Neural Cells from Human Developing Cranial Face Tissue

**Figure 6:**
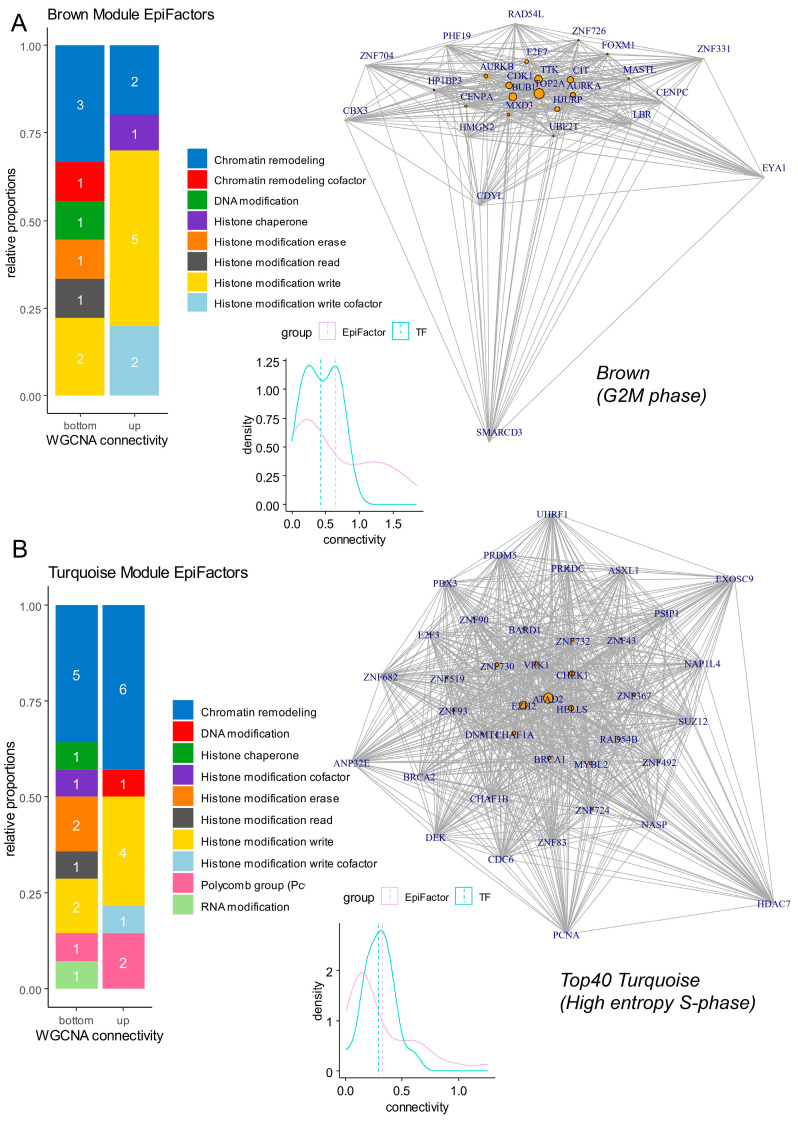
Networks and connectivity of EpiFactors in gene modules associated with cell cycle proliferation phases. (**A**) Network and connectivity focused on EpiFactors in the “brown” gene module associated with the G2M cell cycle phase (numbers in barplots represent the numbers of molecules by class). (**B**) Network and connectivity focused on EpiFactors in the “turquoise” gene module associated with the S cell cycle phase (numbers in barplots represent the numbers of molecules by class).

Inside the proliferative modules (brown and turquoise), gene connectivity was computed at the intra-module level. In the brown module associated with the G2M phase, a high quantification of connectivity was found that was preponderant for EpiFactors (density plot, Figure 6A and Table 1). In the brown module, EpiFactors identified with high connectivity (barplot, Figure 6A) were found to be mainly associated with chromatin remodeling, like TOP2A (network, Figure 6A), and histone modifications (barplot, Figure 6A), like BUB1, CDK1, AURKA, and AURKB (network, Figure 6A), all known as proliferative markers. During the G2M phase in the brown module, very few heterogenous transcription factors were found enriched (Figure 5B). In the turquoise module associated with the S phase of the cell cycle (Figure 6B and Table 2), high connectivity was found that was preponderant for epigenetic factors (density plot, Figure 6B). Epigenetic factors with a high level of connectivity during the S phase were particularly implicated in chromatin remodeling (barplot, Figure 6B), including ATAD2 (ATPase family AAA domain containing 2), HELLS (helicase, lymphoid specific), CHAF1A (chromatin assembly factor 1 subunit A), RAD54B (RAD54 homolog B), PSIP1 (PC4 and SFRS1 interacting protein 1), and CHAF1B (chromatin assembly factor 1 subunit B) (network, Figure 6B and Table 2). Among epigenetic factors enriched during the S phase in the turquoise module, it is interesting to observe that the EZH2 polycomb member and BRCA1 (BRCA1 DNA repair associated) were implicated in histone modifications and presented a high level of connectivity (Table 2). During the S phase, a high representation of transcription factors from the ZNF family (Figure S6C) was found in the network of the turquoise module (Figure 6B and Table 2).

### 3.7. Transcription Hub Regulation during Differentiation Phases of Neural Cells from Human Developing Cranial Face Tissue

**Figure 7:**
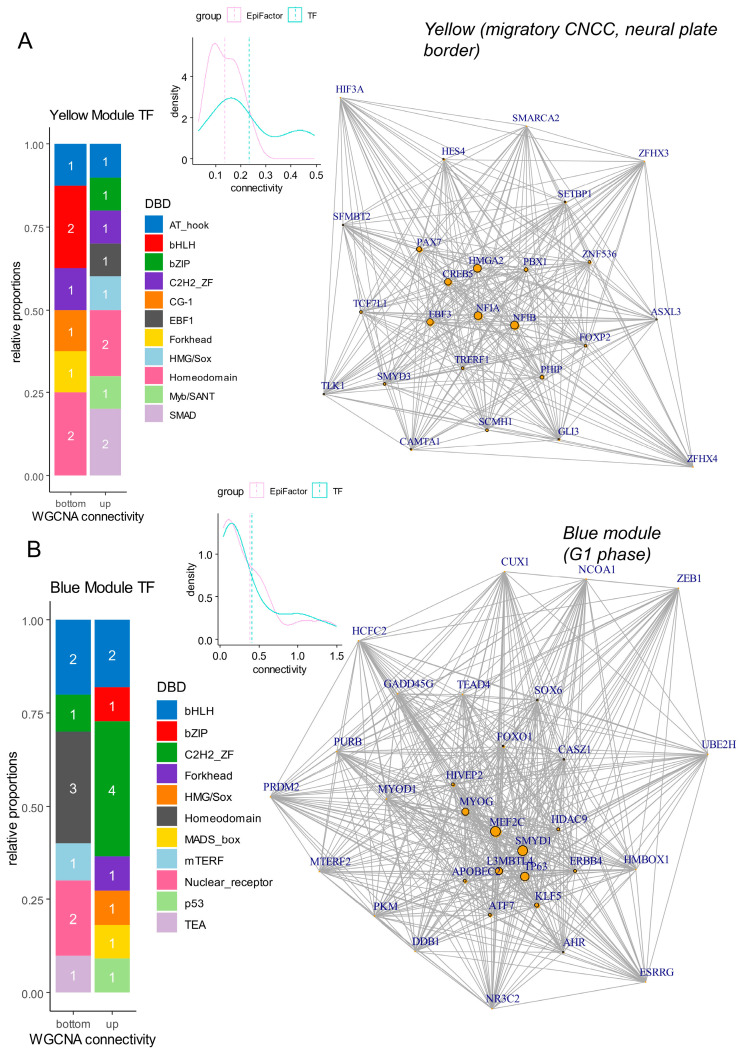
Networks and connectivity of transcription factors in gene modules associated with differentiation processes. (**A**) Network and connectivity focused on transcription factors in the “yellow” gene module associated with neural plate border and migratory CNCC differentiation (numbers in barplots represent the numbers of molecules by class). (**B**) Network and connectivity focused on transcription factors in the “blue” gene module associated with the G1 phase of the cell cycle (numbers in barplots represent the numbers of molecules by class).

Inside differentiation modules (yellow: neural plate border, blue: epidermis), gene connectivity was computed at the intra-module level. During neural plate border differentiation (yellow module), a high connectivity of transcription factors was observed (density plot, Figure 7A). Transcription factors enriched with high connectivity were found mainly harboring SMAD and homeodomain DNA binding domains (DBD) (barplot, Figure 7A) (SMAD: NFIA (nuclear factor IA), NFIB (nuclear factor IB)) (homeodomains: PAX7, PBX1 (PBX homeobox 1)) (network, Figure 7A and Table 3). For cells in the G1 phase with low pseudotime (blue module), there was also a domination of transcription factors with high connectivity (density plot, Figure 7B). In the blue module, transcription factors with high connectivity harbored C2H2_ZF DNA binding domains (barplot, Figure 7B and Table 4) represented by L3MBTL4 (L3MBTL histone methyl-lysine binding protein 4), KLF5 (Kruppel-like factor 5), HIVEP2 (HIVEP zinc finger 2), and CASZ1 (castor zinc finger 1) (network, Figure 7B and Table 4). The MEF2C (myocyte enhancer factor 2C) transcription factor was found to be central to this G1 phase blue module (Figure 7B).

## 4. Discussion

In this study, we explored the intricate balance between transcriptional and epigenetic regulation during the developmental proliferation of human cranial neural crest cells (CNCCs). Our findings provide valuable insights into the dynamic interplay of these regulatory mechanisms across different cell cycle phases and their implications for human craniofacial development and associated human pathologies.

Through our analysis, we identified four distinct cell clusters based on their cell cycle phases: G1, S, G2M, and a mixed cluster. We observed a correlation between cell proliferation and transcriptional regulation, with proliferative cells (G2M and S phases) exhibiting higher entropy compared to non-proliferative cells (G1 phase). This increased entropy in proliferative cells indicates a more diverse and dynamic transcriptional landscape, likely reflecting the heightened regulatory complexity required for cell cycle progression.

Our pseudotime analysis revealed that cells with the highest pseudotime values, indicative of more advanced developmental stages, also exhibited the highest entropy. These cells predominantly occupied the proliferative phases of the cell cycle. This correlation underscores the role of transcriptional plasticity in facilitating the rapid and diverse implementation of cell responses necessary for proliferation and differentiation.

We demonstrated that the pseudotime and associated transcriptional entropy are tightly regulated by epigenetic mechanisms. Disruptions in this epigenetic regulation during embryogenesis can probably lead to significant cell cycle defects, which may manifest in adulthood as craniofacial abnormalities or cancers such as melanoma and neuroblastoma. These findings highlight the critical role of epigenetic control in maintaining the proper balance of cell proliferation and differentiation during development.

Our data showed that CNCCs predominantly reside in the G2M phase, which is closely linked to their migratory capabilities essential for forming various anatomical structures, including the nervous system, melanocytes, neural plate border, and mesoderm. This phase-specific localization emphasizes the importance of the G2M phase in enabling CNCCs to fulfill their migratory and differentiative roles during craniofacial development.

In contrast, epidermal cells and chondrocytes were primarily found in the G1 phase, suggesting a distinct relationship between their differentiation states and proliferative trajectories. This distinction further supports the notion that proper cell cycle regulation is crucial for the accurate formation of cranial tissues. Any aberrations in this regulation could lead to developmental defects, underscoring the need for precise control mechanisms during early developmental stages.

During neural plate border differentiation, we found that PAX7, a well-known important transcription factor (TF) for CNCC differentiation [59], plays a critical role. It is known that Elk3 is essential for the progression from progenitor to definitive neural crest cell. Hence, the relationship between PAX7 and Elk3 is crucial, as Elk3 facilitates the transition of Pax7+ precursors into definitive neural crest cells by enabling the upregulation of neural crest specifier genes such as FoxD3, Sox10, and Snail2 [59]. Loss of the Elk3 protein results in the retention of Pax7+ precursors in the dorsal neural tube, indicating that Elk3′s function is necessary for the proper differentiation pathway initiated by PAX7. Furthermore, PAX7 was found to be associated with another homeodomain protein, PBX1 [60]. Loss of Pbx in cranial neural crest cells, unlike in epithelium, results in cleft palate only and a broader midface, highlighting the tissue-specific roles of these factors in craniofacial development.

Moreover, PAX7 and Elk3 are significant in the context of melanoma and neuroblastoma, both of which originate from neural crest-derived cells. In melanoma, PAX7 dysregulation can influence melanocyte development and transformation [61]. Since melanocytes share a common embryonic origin with neural crest cells, pathways involving PAX7 and Elk3 may play a role in melanoma progression. Abnormal PAX7 activity could lead to unchecked proliferation and the survival of melanocyte precursors, contributing to melanoma [62]. Elk3, which regulates the transition of progenitors to definitive neural crest cells, might similarly influence melanocyte dynamics and melanoma development [63]. On the other hand, neuroblastoma, a cancer originating from neural crest-derived cells in the sympathetic nervous system, is also related to the roles of PAX7 and Elk3. The dysregulation of PAX7 can affect neuroblastoma cell behavior, and the retention of Pax7+ precursors due to Elk3 loss suggests that disruptions in these pathways could contribute to neuroblastoma pathology, where progenitor cells fail to differentiate properly [64]. The inability to upregulate neural crest specifier genes like FoxD3, Sox10, and Snail2, essential for cell migration and differentiation, might result in the persistence of immature, proliferative cells contributing to tumorigenesis in neuroblastoma [65].

Conjointly, for PAX7 and PBX1 homeodomain transcription factors during neural plate border differentiation, a central network was formed with SMAD domain transcription factors represented by NFIA and NFIB. In pathology, NFIA and NFIB are known to induce cellular differentiation in high-grade astrocytoma [66]. In the context of premature neurogenesis and abnormal neuronal migration in the mutant brain with altered embryonic neural stem and progenitor cell homeostasis, HDAC3 has been shown to interact with NFIB to perform transcriptional regulation [67]. NFIB drives astrocytic maturation within the developing spinal cord [68].

During neural crest differentiation, the transcription factor EBF3 was found with centrality in the network of transcription factors. EBF3 is known to intervene as a regulator of neural differentiation of during primary neurogenesis in Xenopus [69]. HMGA2 was also found with high centrality in neural plate border differentiation and is known to be required for neural crest cell specification in *Xenopus laevis* [70]. During pathology at the multicentric level, HMGA2 gene rs8756 A>C polymorphism has been linked to a reduced risk of neuroblastoma in Chinese children [71].

To conclude, the alternative balance between transcriptional and epigenetic regulation observed in our study has significant implications for understanding human pathologies. Craniofacial syndromes and cancers, such as melanoma and neuroblastoma, may arise from the dysregulation of these intricate mechanisms. Our results emphasize the necessity of maintaining a harmonious interplay between transcriptional and epigenetic factors to ensure proper cranial development and prevent disease.

## 5. Conclusions

In conclusion, our study provides a comprehensive view of the regulatory dynamics underlying the developmental proliferation of human CNCCs. The identified balance between transcriptional and epigenetic regulation is crucial for proper cell cycle progression and differentiation. Disruptions in these processes can lead to developmental defects and pathological conditions, highlighting the importance of these mechanisms in both normal development and disease. Future research should focus on further elucidating the specific epigenetic modifications and transcriptional networks involved in CNCC regulation to develop targeted therapeutic strategies for related human pathologies.

## Figures and Tables

**Table 1:** Network connectivity of epigenetic/transcription factors identified in the brown module associated with the G2M phase of the cell cycle (TF: transcription factor).

Gene	Group	Subgroup	Connectivity	Association
TOP2A	EpiFactor	Chromatin remodeling	1.846	G2M phase
BUB1	EpiFactor	Histone modification write	1.538	G2M phase
TTK	EpiFactor	Histone modification write cofactor	1.481	G2M phase
CDK1	EpiFactor	Histone modification write	1.355	G2M phase
CIT	EpiFactor	Histone modification write cofactor	1.241	G2M phase
AURKA	EpiFactor	Histone modification write	1.059	G2M phase
HJURP	EpiFactor	Histone chaperone	0.966	G2M phase
AURKB	EpiFactor	Histone modification write	0.893	G2M phase
E2F7	TF	E2F	0.723	G2M phase
MXD3	TF	bHLH	0.637	G2M phase
MASTL	EpiFactor	Histone modification write	0.473	G2M phase
HP1BP3	EpiFactor	Chromatin remodeling	0.432	G2M phase
FOXM1	TF	Forkhead	0.387	G2M phase
UBE2T	EpiFactor	Histone modification write	0.312	G2M phase
ZNF726	TF	C2H2_ZF	0.311	G2M phase
CENPC	EpiFactor	DNA modification	0.271	G2M phase
HMGN2	EpiFactor	Chromatin remodeling	0.249	G2M phase
PHF19	EpiFactor	Chromatin remodeling	0.231	G2M phase
RAD54L	EpiFactor	Chromatin remodeling	0.175	G2M phase
ZNF704	TF	C2H2_ZF	0.144	G2M phase
ZNF331	TF	C2H2_ZF	0.139	G2M phase
CBX3	EpiFactor	Histone modification read	0.110	G2M phase
CDYL	EpiFactor	Histone modification write	0.036	G2M phase
EYA1	EpiFactor	Histone modification erase	0.029	G2M phase
SMARCD3	EpiFactor	Chromatin remodeling cofactor	0.002	G2M phase

**Table 2:** Network connectivity of epigenetic/transcription factors identified in the turquoise module associated with the S phase of the cell cycle (TF: transcription factor).

Gene	Group	Subgroup	Connectivity	Association
ATAD2	EpiFactor	Chromatin remodeling	1.262	S phase
EZH2	EpiFactor	Polycomb group (PcG) protein	0.967	S phase
CHEK1	EpiFactor	Histone modification write	0.761	S phase
HELLS	EpiFactor	Chromatin remodeling	0.683	S phase
CHAF1A	EpiFactor	Chromatin remodeling	0.616	S phase
ZNF730	TF	C2H2_ZF	0.597	S phase
BRCA1	EpiFactor	Histone modification write cofactor	0.592	S phase
VRK1	EpiFactor	Histone modification write	0.590	S phase
ZNF732	TF	C2H2_ZF	0.448	S phase
BARD1	EpiFactor	Histone modification write	0.430	S phase
DNMT1	EpiFactor	DNA modification	0.412	S phase
DNMT1	TF	CxxC	0.412	S phase
MYBL2	TF	Myb/SANT	0.406	S phase
RAD54B	EpiFactor	Chromatin remodeling	0.396	S phase
ZNF367	TF	C2H2_ZF	0.360	S phase
ZNF93	TF	C2H2_ZF	0.353	S phase
ZNF43	TF	C2H2_ZF	0.315	S phase
ZNF90	TF	C2H2_ZF	0.314	S phase
ZNF519	TF	C2H2_ZF	0.301	S phase
ZNF724	TF	C2H2_ZF	0.298	S phase
ZNF492	TF	C2H2_ZF	0.240	S phase
E2F3	TF	E2F	0.225	S phase
PSIP1	EpiFactor	Chromatin remodeling	0.219	S phase
PRKDC	EpiFactor	Histone modification write	0.203	S phase
CHAF1B	EpiFactor	Chromatin remodeling	0.195	S phase
PBX3	TF	Homeodomain	0.189	S phase
ASXL1	EpiFactor	Polycomb group (PcG) protein	0.188	S phase
ZNF682	TF	C2H2_ZF	0.187	S phase
BRCA2	EpiFactor	Histone modification write	0.182	S phase
SUZ12	EpiFactor	Polycomb group (PcG) protein	0.164	S phase
ZNF83	TF	C2H2_ZF	0.157	S phase
NASP	EpiFactor	Chromatin remodeling	0.157	S phase
NAP1L4	EpiFactor	Histone modification cofactor	0.149	S phase
CDC6	EpiFactor	Chromatin remodeling	0.149	S phase
PRDM5	EpiFactor	Histone modification write	0.149	S phase
PRDM5	TF	C2H2_ZF	0.149	S phase
DEK	EpiFactor	Chromatin remodeling	0.131	S phase
UHRF1	EpiFactor	Histone modification read	0.119	S phase
EXOSC9	EpiFactor	RNA modification	0.112	S phase
ANP32E	EpiFactor	Histone chaperone	0.093	S phase
PCNA	EpiFactor	Chromatin remodeling	0.086	S phase
HDAC7	EpiFactor	Histone modification erase	0.035	S phase
ZRANB3	EpiFactor	Chromatin remodeling	0.021	S phase
EYA4	EpiFactor	Histone modification erase	0.003	S phase
ZNF395	TF	C2H2_ZF	0.003	S phase

**Table 3:** Network connectivity of epigenetic/transcription factors identified in the yellow module associated with neural plate border differentiation (TF: transcription factor).

Gene	Group	Subgroup	Connectivity	Association
NFIA	TF	SMAD	0.494	neural plate border
NFIB	TF	SMAD	0.487	neural plate border
HMGA2	TF	AT_hook	0.441	neural plate border
EBF3	TF	EBF1	0.420	neural plate border
CREB5	TF	bZIP	0.417	neural plate border
PAX7	TF	Homeodomain	0.356	neural plate border
PBX1	TF	Homeodomain	0.247	neural plate border
PHIP	EpiFactor	Histone modification read	0.232	neural plate border
ZNF536	TF	C2H2_ZF	0.206	neural plate border
TRERF1	TF	Myb/SANT	0.188	neural plate border
TCF7L1	TF	HMG/Sox	0.186	neural plate border
SCMH1	EpiFactor	Polycomb group (PcG) protein	0.185	neural plate border
FOXP2	TF	Forkhead	0.171	neural plate border
SMYD3	EpiFactor	Histone modification write	0.157	neural plate border
SETBP1	TF	AT_hook	0.154	neural plate border
GLI3	TF	C2H2_ZF	0.142	neural plate border
HES4	TF	bHLH	0.142	neural plate border
CAMTA1	TF	CG-1	0.133	neural plate border
ASXL3	EpiFactor	Polycomb group (PcG) protein	0.101	neural plate border
SFMBT2	EpiFactor	Polycomb group (PcG) protein	0.094	neural plate border
TLK1	EpiFactor	Histone modification write	0.090	neural plate border
SMARCA2	EpiFactor	Histone modification read	0.060	neural plate border
ZFHX3	TF	Homeodomain	0.054	neural plate border
HIF3A	TF	bHLH	0.044	neural plate border
ZFHX4	TF	Homeodomain	0.030	neural plate border

**Table 4:** Network connectivity of epigenetic/transcription factors identified in the blue module associated with the G1 phase (TF: transcription factor).

Gene	Group	Subgroup	Connectivity	Network
MEF2C	TF	MADS_box	1.492	epidermis
SMYD1	EpiFactor	Histone modification write	1.383	epidermis
TP63	TF	p53	1.266	epidermis
L3MBTL4	EpiFactor	Histone modification read	1.041	epidermis
L3MBTL4	TF	C2H2_ZF	1.041	epidermis
MYOG	TF	bHLH	1.005	epidermis
KLF5	TF	C2H2_ZF	0.802	epidermis
ATF7	TF	bZIP	0.604	epidermis
APOBEC2	EpiFactor	DNA modification	0.590	epidermis
HIVEP2	TF	C2H2_ZF	0.530	epidermis
ERBB4	EpiFactor	Histone modification cofactor	0.482	epidermis
HDAC9	EpiFactor	Histone modification erase	0.481	epidermis
FOXO1	TF	Forkhead	0.371	epidermis
AHR	TF	bHLH	0.282	epidermis
CASZ1	TF	C2H2_ZF	0.251	epidermis
SOX6	TF	HMG/Sox	0.240	epidermis
MYOD1	TF	bHLH	0.213	epidermis
HMBOX1	TF	Homeodomain	0.179	epidermis
TEAD4	TF	TEA	0.144	epidermis
DDB1	EpiFactor	Histone modification write	0.138	epidermis
GADD45G	EpiFactor	Chromatin remodeling	0.135	epidermis
NR3C2	TF	Nuclear_receptor	0.120	epidermis
PKM	EpiFactor	Histone modification write cofactor	0.113	epidermis
ESRRG	TF	Nuclear_receptor	0.097	epidermis
NCOA1	EpiFactor	Histone modification write	0.090	epidermis
NCOA1	TF	bHLH	0.090	epidermis
HCFC2	EpiFactor	Histone modification write cofactor	0.090	epidermis
MTERF2	TF	mTERF	0.067	epidermis
CUX1	TF	Homeodomain	0.059	epidermis
PRDM2	EpiFactor	Histone modification write	0.049	epidermis
PRDM2	TF	C2H2_ZF	0.049	epidermis
UBE2H	EpiFactor	Histone modification write	0.046	epidermis
ZEB1	TF	Homeodomain	0.045	epidermis

## Data Availability

Availability for bio-informatics codes: https://github.com/cdesterke/CNCC_scripts (accessed on 18 July 2024).

## Supplementary Materials

The following supporting information can be downloaded at https://www.mdpi.com/article/10.3390/cells13191634/s1, Figure S1: Seurat cluster stratification of neural cells from human developing cranial face tissues (CS17); Figure S2: Best ten markers identified in low-pseudotime neural cells; Figure S3: Best ten markers identified in high-pseudotime neural cells; Figure S4: Pseudotime expression of representative markers identified as regulated along neural cell trajectory; Figure S5: WGCNA adjacent matrix and transcription factor stratification for brown and turquoise modules. Table S1: Significant genes found on pseudotime trajectory of human developing cranial neural crest cells (CS17). Table S1 is available online at the following address: https://figshare.com/s/9cc9c4559b144b32704b (accessed on 19 July 2024), DOI: 10.6084/m9.figshare.26334847.
