# Correction: Desterke et al. Alternative Balance between Transcriptional and Epigenetic Regulation during Developmental Proliferation of Human Cranial Neural Crest Cells. *Cells* 2024, *13*, 1634

**DOI:** 10.3390/cells14181419

**Published:** 2025-09-11

**Authors:** Christophe Desterke, Raquel Francés, Claudia Monge, Agnès Marchio, Pascal Pineau, Jorge Mata-Garrido

**Affiliations:** 1 Faculté de Médecine du Kremlin Bicêtre, Université Paris-Saclay and INSERM UMRS1310, 94270 Le Kremlin-Bicêtre, France; christophe.desterke@inserm.fr; 2 Cell and Tissue Biology Group, Anatomy and Cell Biology Department, University of Cantabria-IDIVAL, 39011 Santander, Spain; 3 International Joint Laboratory of Molecular Anthropological Oncology (LOAM), IRD, INEN, Lima 15038, Peru

## Funding

In the original publication [1], the funder “MEAE AMBASS FRANCE AU PEROU FSPI—S-AC23007, Filière Santé Maladie Rare «TeteCou-CRANIOST»” must be modified by “This research received no external funding” due to the fact that, since it is a computational-developed article, the use of funds was not necessary.

## Affiliations

In the published publication [1], there was an error regarding the affiliations for Raquel Francés, Claudia Monge, Agnès Marchio, Pascal Pineau and Jorge Mata-Garrido. As a replacement for affiliations 2 and 3, as well as the correspondence email, the updated affiliations should include the following:

^2^  Cell and Tissue Biology Group, Anatomy and Cell Biology Department, University of Cantabria-IDIVAL, 39011 Santander, Spain
^3^  International Joint Laboratory of Molecular Anthropological Oncology (LOAM), IRD, INEN, Lima 15038, Peru

In addition, the corresponding author, Jorge Mata-Garrido, moves from affiliation 3 to the new affiliation 2.

* Correspondence: jmatag@unican.es

All emails, except for christophe.desterke@inserm.fr and for the correspondence email, must, in turn, be removed from the article.

The authors state that the scientific conclusions are unaffected. This correction was approved by the Academic Editor. The original publication has also been updated.
