# Supplementary material for: Alternative Balance between Transcriptional and Epigenetic Regulation during Developmental Proliferation of Human Cranial Neural Crest Cells
Source: Cells. 2024 Sep 30;13(19):1634. doi: 10.3390/cells13191634 (PMC11476078; doi:10.3390/cells13191634)
Supplement: Supplementary file 1 [file cells-13-01634-s001.zip › cells-3209353-supplementary.pdf]

cells

MDPI

(SUPPLEMENTARY DATA)

Article

Alternative balance between transcriptional and epigenetic regulation during developmental proliferation of human cranial neural crest cells

Chirstophe Desterke[1], Raquel Francés[2,†], Claudia Monge[3,†], Àgnes Marchio[3], Pascal Pineau[3] & Jorge Mata-Garrido[3,*]

[1] Faculté de Médecine du Kremlin Bicetre, University Paris-Sud, Université Paris-Saclay, Le Kremlin-Bicêtre, France; christophe.desterke@inserm.fr
[2] Energy & Memory, Brain Plasticity Unit, CNRS, ESPCI Paris, PSL Research University, Paris, France; raquel.frances@espci.fr
[3] Unité Organisation Nucléaire et Oncogenèse, INSERM U993, Institut Pasteur, Paris, France; claudia.monge@pasteur.fr; agnes.marchio@pasteur.fr; pascal.pineau@pasteur.fr.
† Contributed equally.
* Correspondence: jorge.mata-garrido@pasteur.fr

Supplemental Figures

Figure S1: Seurat cluster stratification of neural cells from human developing cranial face tissues (CS17):A/ Principal component analysis on scRNAseq of neural cell subtype with projection of seurat clusters; B/Barplot of cell proportion stratified by seurat cluster identity and cell cycle phase prediction (chi.square test p-value between seurat cluster identity and cell phase prediction); C/ Single cell expression heatmap with best markers identified by Seurat clusters.

Figure S2: Best ten markers identified in low pseudotime neural cells: Pseudotime expression plot of 10 markers for cells with lowest pseudotime value by TSCAN analysis on neural cell subtype

Figure S3: Best ten markers identified in high pseudotime neural cells: Pseudotime expression plot of 10 markers for cells with highest pseudotime value by TSCAN analysis on neural cell subtype

Figure S4: Pseudotime expression of representative markers identified as regulated along neural cell trajectory: A/ Pseudotime expression with cell cycle phase decomposition for representative markers found on trajectory; B/ Pseudotime expression with cell cycle phase decomposition for epigenetic markers found on trajectory; C/ Pseudotime expression with cell cycle phase decomposition for cell cycle markers found on trajectory

Figure S5: WGCNA adjacent matrix and transcription factors stratification brown and turquoise modules; Table S1: Table of pseudotime results: A/Heatmap of WGCNA adjacent matrix with gene module identification (yellow, brown, blue, and turquoise; B/ Barplot of transcription factors enriched in brown module stratified on connectivity threshold; C/ Barplot of transcription factors enriched in turquoise module stratified on connectivity threshold (DBD: DNA binding domain)

Figure S1

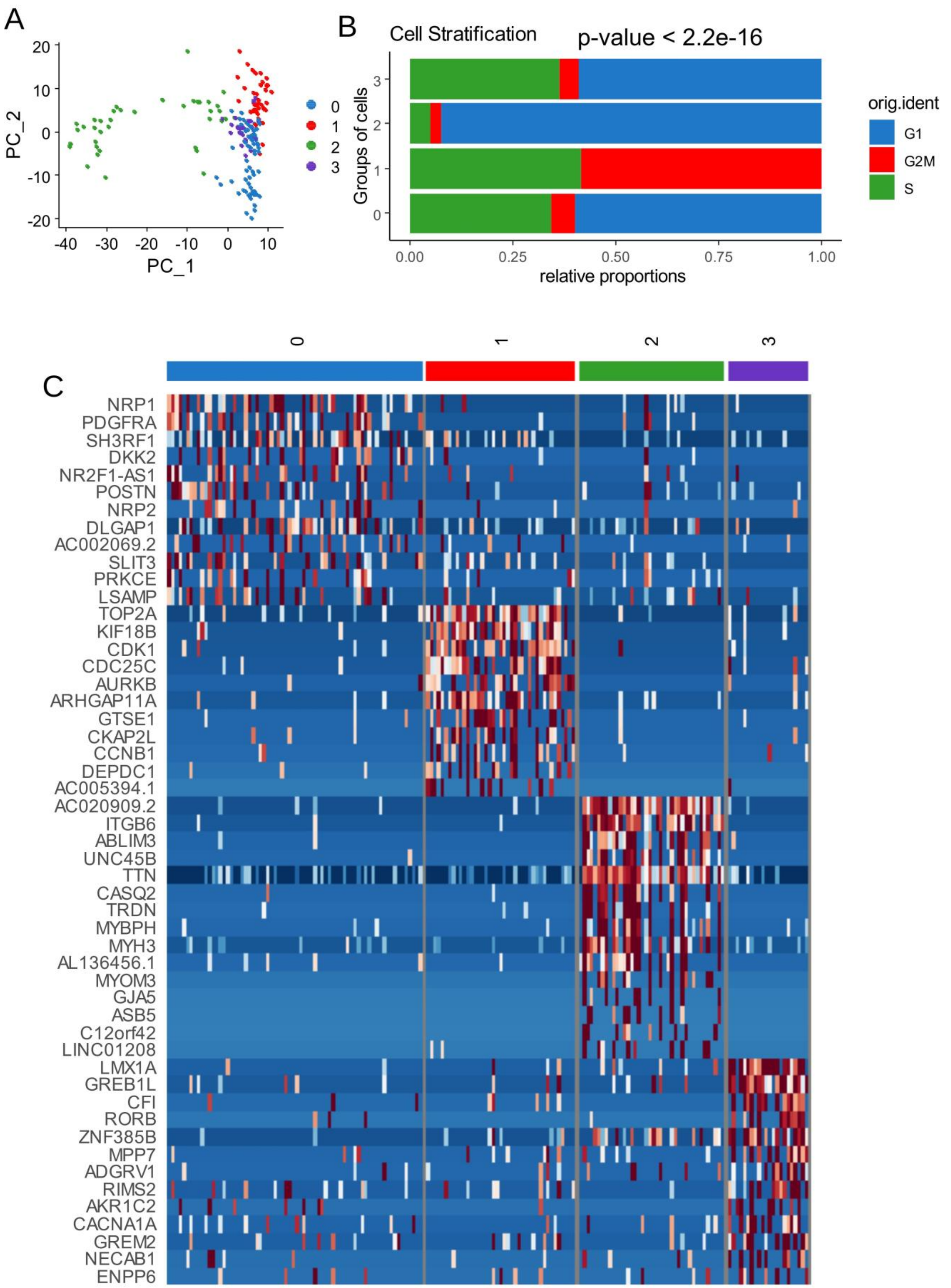
A
PC_2
PC_1
0
1
2
3
B
Cell Stratification
p-value < 2.2e-16
Groups of cells
relative proportions
orig.ident
G1
G2M
S
C
NRP1
PDGFRA
SH3RF1
DKK2
NR2F1-AS1
POSTN
NRP2
DLGAP1
AC002069.2
SLIT3
PRKCE
LSAMP
TOP2A
KIF18B
CDK1
CDC25C
AURKB
ARHGAP11A
GTSE1
CKAP2L
CCNB1
DEPDC1
AC005394.1
AC020909.2
ITGB6
ABLIM3
UNC45B
TTN
CASQ2
TRDN
MYBPH
MYH3
AL136456.1
MYOM3
GJA5
ASB5
C12orf42
LINC01208
LMX1A
GREB1L
CFI
RORB
ZNF385B
MPP7
ADGRV1
RIMS2
AKR1C2
CACNA1A
GREM2
NECAB1
ENPP6

Figure S2

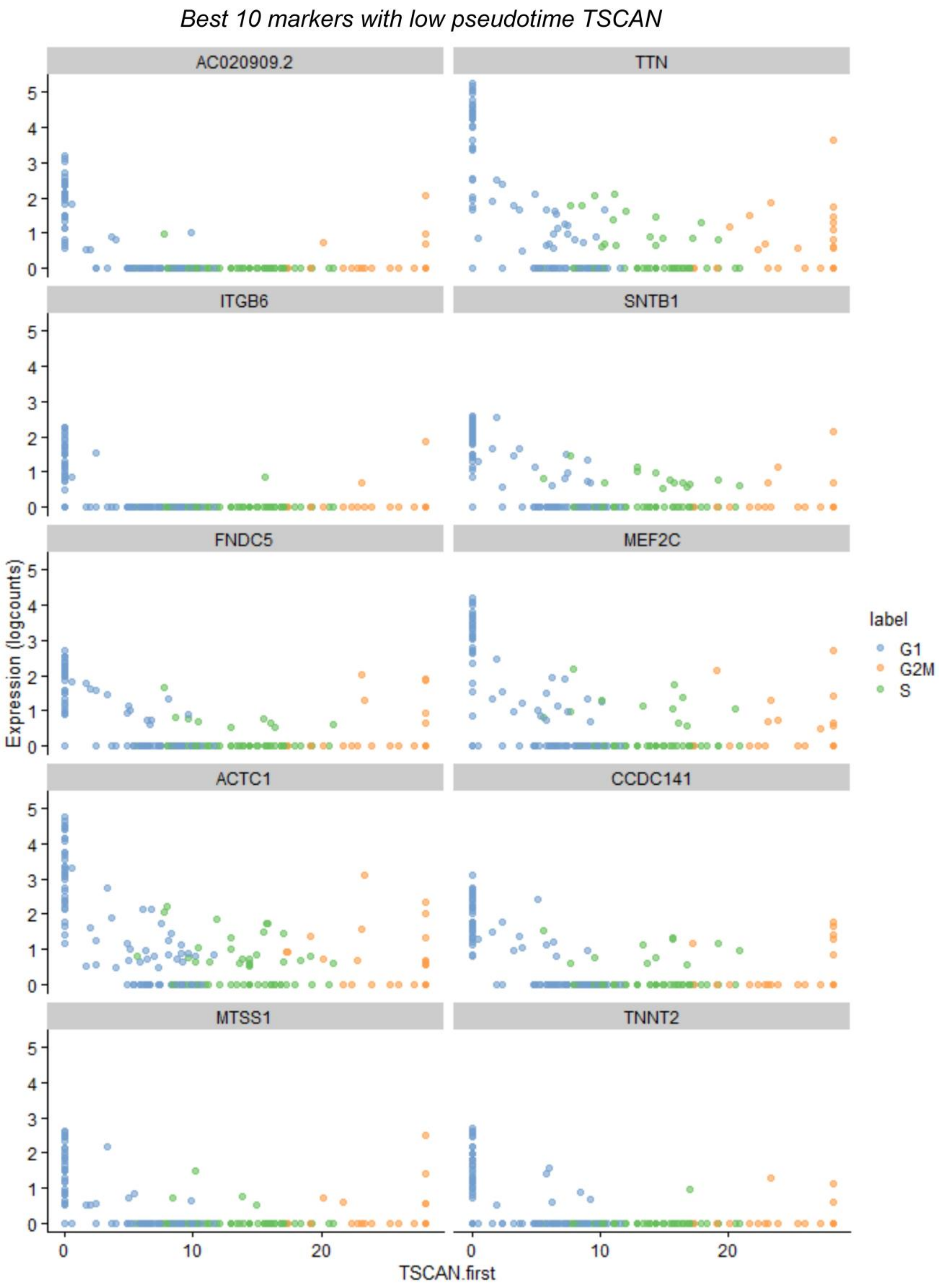
Best 10 markers with low pseudotime TSCAN
AC020909.2
TTN
ITGB6
SNTB1
FNDC5
MEF2C
ACTC1
CCDC141
MTSS1
TNNT2
Expression (logcounts)
TSCAN.first
label
G1
G2M
S
0
1
2
3
4
5
0
10
20

Figure S3

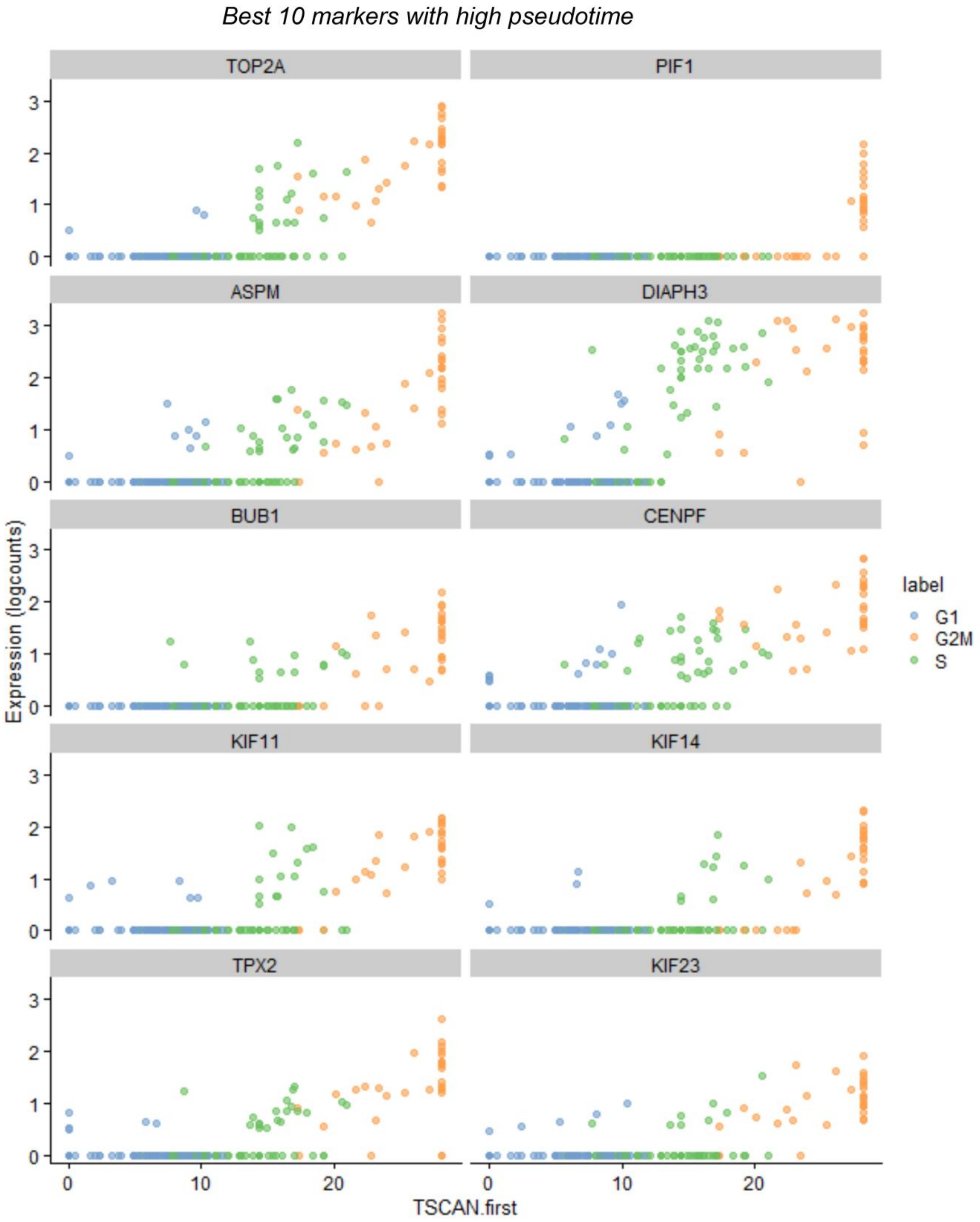
Best 10 markers with high pseudotime
TOP2A
PIF1
ASPM
DIAPH3
BUB1
CENPF
KIF11
KIF14
TPX2
KIF23
Expression (logcounts)
TSCAN.first
label
G1
G2M
S
0
1
2
3
0
10
20

Figure S4

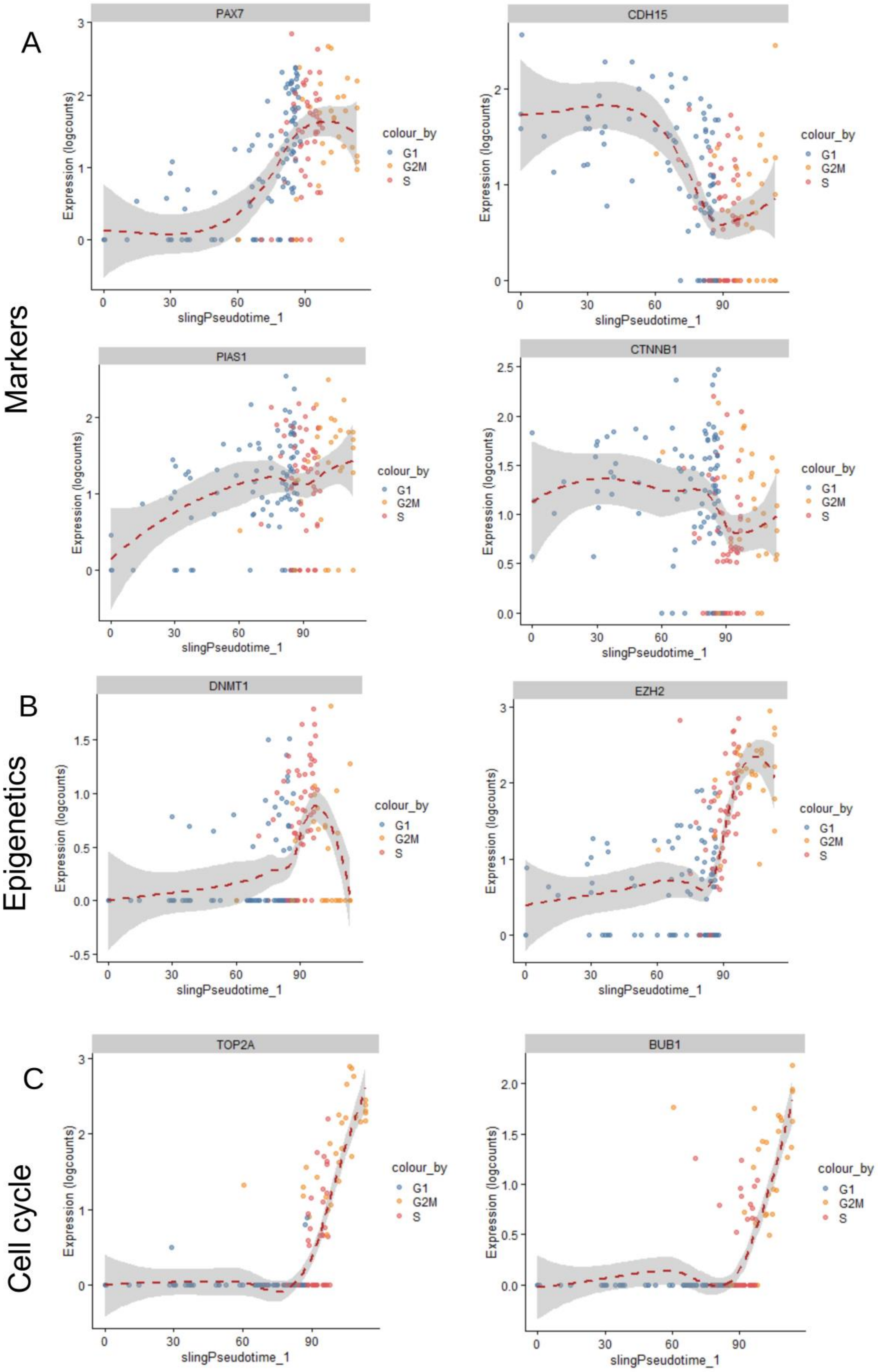
A
Markers
PAX7
CDH15
PIAS1
CTNNB1
B
Epigenetics
DNMT1
EZH2
C
Cell cycle
TOP2A
BUB1
Expression (logcounts)
slingPseudotime_1
colour_by
G1
G2M
S

Figure S5

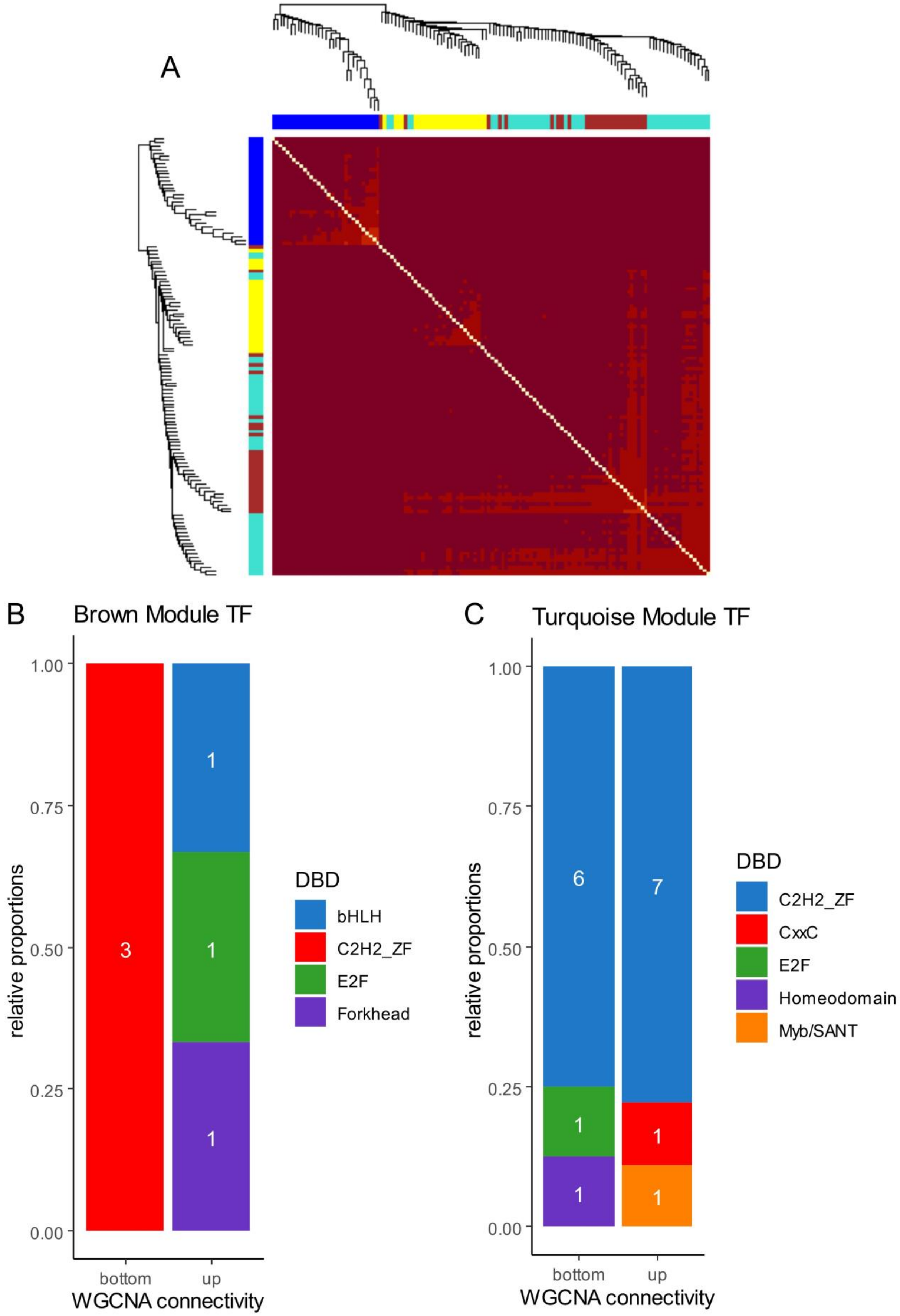
A
B
Brown Module TF
1.00
0.75
0.50
0.25
0.00
relative proportions
3
1
1
1
bottom
up
WGCNA connectivity
DBD
bHLH
C2H2_ZF
E2F
Forkhead
C
Turquoise Module TF
1.00
0.75
0.50
0.25
0.00
relative proportions
6
7
1
1
1
1
bottom
up
WGCNA connectivity
DBD
C2H2_ZF
CxxC
E2F
Homeodomain
Myb/SANT
